# Occupational exposures and respiratory symptoms and lung function among hairdressers in Iran: a cross-sectional study

**DOI:** 10.1007/s00420-020-01645-z

**Published:** 2021-01-18

**Authors:** Behzad Heibati, Maritta S. Jaakkola, Taina K. Lajunen, Alan Ducatman, Zahra Bamshad, Samira Eslamizad, Fatemeh Shafee, Ali Karimi, Jouni J. K. Jaakkola

**Affiliations:** 1 Center for Environmental and Respiratory Health Research, Faculty of Medicine, University of Oulu, Aapistie 5B, P.O. Box 5000, 90014 Oulu, Finland; 2 Faculty of Medicine, Biocenter Oulu, University of Oulu, P.O. Box 5000, 90014 Oulu, Finland; 3 Medical Research Center Oulu, Oulu University Hospital, University of Oulu, P.O. Box 8000, 90014 Oulu, Finland; 4 West Virginia University School of Public Health, Morgantown, WV USA; 5 Department of Occupational Health, School of Public Health, Shiraz University of Medical Sciences, Shiraz, Iran; 6 Food Safety Research Center, Shahid Beheshti University of Medical Sciences, Tehran, Iran; 7 Department of Occupational Health, School of Public Health, Tehran University of Medical Sciences, Tehran, Iran; 8 Finnish Meteorological Institute, P.O. Box 503, 00101 Helsinki, Finland

**Keywords:** Occupational exposures, Hairdressing, Lung function, Spirometry, Respiratory symptoms

## Abstract

**Objective:**

Exposures at hairdressers’ work have been reported to lead to an increased risk of several health outcomes. The present study aimed to investigate the relations between occupational exposures and respiratory symptoms and lung function among hairdressers in Iran.

**Methods:**

We conducted a cross-sectional study to compare potential respiratory effects among 140 women working as hairdressers to such effects among 140 women working as office workers (administrative personnel). Both groups worked in Shiraz, Iran. Respiratory symptoms were assessed by a standard respiratory questionnaire. The questionnaire also inquired about substances used and workspace conditions, including ventilation type. Lung function levels were measured by spirometry.

**Results:**

Respiratory symptoms, including cough, wheezing, shortness of breath, and chest tightness were significantly more frequent in hairdressers compared to the reference group (*p* < 0.05). After controlling for potential confounders, hairdressers had a prevalence ratio (PR) of 2.18 (95% CI 1.26–3.77) for cough, 9.59 (95% CI 1.004–91.73) for wheezing, 2.06 (95% CI 1.25–3.39) for shortness of breath, and 3.31 (95% CI 1.84–5.97) for chest tightness compared to the reference group. Lung function parameters (including VC, FVC, and FEV1) were significantly reduced in hairdressers (*p* < 0.001). Absence of air conditioning predicted greater reduction in lung function (*p* < 0.05) in the exposed. Decrease in FVC with normal FEV1/FVC in the exposed group suggested existence of restrictive lung function.

**Conclusions:**

This study provides evidence of increased prevalence of respiratory symptoms and restrictive lung function impairment among hairdressers in Iran.

**Supplementary Information:**

The online version contains supplementary material available at 10.1007/s00420-020-01645-z.

## Introduction

Hairdressers’ work environment has been reported to contain exposures that can be harmful for reproductive health and can cause cancer, skin irritation, and allergic diseases (Quiros-Alcala et al. 2019). Quiros-Alcala et al. reviewed the literature published from 2014 to 2019 on hair and nail salon workers. They concluded that there is consistent evidence that working in hair and nail salons may increase the risk of respiratory effects. (Quiros-Alcala et al. 2019). Potentially hazardous exposures include, but are not limited to, bleaches, conditioners, detergents, dyes, fixatives, and relaxers or straighteners that are most often used as commercially prepared mixtures (Labrèche et al. 2003; Pak et al. 2013). Several of these product categories can generate chemical aerosols during hairdressing activities, and hairdressing-specific aerosol particles have been shown to be capable to penetrate into the lungs (Nilsson et al. 2016)

Frequently used specific chemicals that have been reported to have adverse effects on people include formaldehyde in shampoos, ammonium compounds in hair dyes and nail cleaners, ammonium acetate, polyvinyl and ethanol in hair sprays, persulfate salt such as sodium persulfate and potassium persulfate in hair bleaches, ammonium, potassium, solvents, and phenylene diamine in hair dyes, glycerol thioglycolate in permanent hair curler, styrene and 1,4 dioxane in hair extension glues, phthalates as fixatives, and hydrogen peroxide in emulsions and creams (Pak et al. 2013; Quiros-Alcala et al. 2019). Hair bleaching and use of hair sprays have been reported to be particularly hazardous work tasks (Leino et al. 1998). During bleaching (i.e., discoloring), hairdressers use a mixture of oxidized powder and an alkaline solution to fade the natural hair color. When the hairdressers mix these substances, some of the chemicals are released into the air and are inhaled by the hairdressers, so they can reach the lining of the airways of the hairdressers and thus can cause respiratory symptoms (Quiros-Alcala et al. 2019). The causal mechanisms can be either an irritant mechanism, or in some cases, a hypersensitivity (i.e., allergic) mechanism. Norwegian researchers measured chemical pollutants in hairdressing salons and reported that the saturation of chemicals, such as ammonia (with concentrations of 0.3–10 mg/m^3^), in salon air can stimulate irritation in the lining of airways during and after bleaching operations (Hollund and Moen 1998). Probably due to cumulative exposures, Norwegian hairdressers aged > 40 years have been reported to have a higher prevalence of some respiratory symptoms, such as wheezing, breathlessness, compared to younger hairdressers and to office workers of the same age (Hollund et al. 2001). The probable explanation for higher prevalence of symptoms in older hairdressers is cumulative work-related exposures. A study from Turkey reported twice as high prevalence of asthma (14.6%) among hairdressers compared to the general population. (Akpinar-Elci et al. 2002). A recent review of hairdressers’ health outcomes has documented that excess of respiratory symptoms, lung function decrements, and markers of lung inflammation are more common among hair salon workers following their employment as hairdressers or compared to control populations, including jobs in education, as well as the unemployed (Quiros-Alcala et al. 2019).

Although increased risk of respiratory diseases has been reported in hairdressing occupation, most of the studies in this field have been conducted in high-income countries (Albin et al. 2002; Mahmoudi 1996; Slater et al. 2000), while only a few studies have been conducted in low and middle-income countries, such as Iran. Two previous easily accessible English-language reports documented problems in hairdressers’ health from the Middle East (Akpinar-Elci et al. 2002; Nemer et al. 2015). Health and safety regulations, as well as hairdressing product preferences may differ across the world and are likely to include more problems in the low-income countries. To fill in this gap in knowledge, we assessed potential effects of occupational exposure among hairdressers in Iran on the occurrence of respiratory symptoms and level of lung function. An additional goal was to raise awareness among the regional practitioners and health authorities to encourage them to take the steps needed to reduce occupational hazards among the hairdressers in this area.

## Methods

### Study population

A group of 140 hairdressers with occupational exposures, working in different areas of a large city Shiraz in Iran (pop. 1,566,000), was recruited based on the following eligibility criteria: (i) having worked for at least an year as a hairdresser, and (ii) no history of any previous respiratory disease or lung surgery, and (iii) no previous history of occupational exposure to hazardous workplace substances. As the unexposed reference group, we recruited 140 female office workers with similar demographic characteristics who were working in the same city of Shiraz. Reference population eligibility criteria were no history of occupational exposure to pollutants in hair salons or other workplace sites. Only women were eligible to participate, as the prevalence of male hairdressers in Iran is extremely low.

### Data collection

Data collection was based on a structured questionnaire and pulmonary functions tests (PFTs).

A trained technician conducted an interview at the same session with the pulmonary function tests (PFTs) for both the exposed and unexposed groups. The questionnaire included information on (1) personal characteristics, (2) previous and current health conditions and current respiratory symptoms, (3) details of current job, including location, working habits, working hours, use of chemicals and other substances, use of personal protective equipment (PPE), potential air conditioning in the workplace, safety training, and availability of health surveillance, and (4) potential confounders and modifiers, such as smoking habits (cigarettes/day), and daily use of water pipe.

### Exposure assessment

In the exposure assessment, two approaches were applied. First, the exposure was assessed on the basis of job category: hairdresser (coded 1) and office worker (coded 0). Second, duration of work in years as a hairdresser was used as a quantitative measure of exposure. Duration was categorized into quartiles  (low ≤ 6 years, medium > 6 and ≤ 10 years, high > 10 and ≤ 15 years, very high > 15 years).

### Outcome assessment

The main outcomes of interest were the occurrence of five respiratory symptoms, including chronic cough, phlegm production, wheezing, shortness of breath, and chest tightness, and four lung function parameters, including vital capacity (VC), forced vital capacity (FVC), forced expiratory volume in one second (FEV_1_) and FEV_1_ to FVC ratio (FEV_1_ to FVC). Both absolute values and percentage predicted values of the lung function parameters were used in the analyses.

Occurrence of respiratory symptoms was inquired in an interview that used the standard respiratory questionnaire form provided by the American Thoracic Society (Ferris 1978). This questionnaire has been standardized in 1978 and used in numerous countries, so its validity and reliability have been confirmed (Ferris 1978).

The spirometer used was Fukuda Sangyo ST-150 (Fukuda, Japan). FVC% predicted and FEV1% predicted were already controlled for sex, age, and height in the prediction equations. These were based on GLI spirometry reference values, i.e., multi-ethnic reference values for spirometry for the 3–95-yr age range (Quanjer et al 2012).

### Statistical analysis

We compared the risk of respiratory symptoms and average levels of lung function parameters between the hairdressers as the exposed group and the office workers as the reference group. We used prevalence ratio (PR) as the measure of the relation between exposure and the risk of respiratory symptoms, including cough, phlegm, productive cough, wheezing, shortness of breath, and tightness in the chest. We adjusted for age, marital status, education, and body mass index, BMI (model 1), and in addition for cigarette smoking and use of waterpipe (model 2) in Poisson regression analysis applying SAS procedure GENMOD, with logarithmic link function. We applied LSMEANS-statement to obtain the effect estimates and their 95% confidence intervals (CI).

We estimated the relations between exposure and lung function levels using multiple linear regression. The analyses of the absolute lung function values (expiratory VC, FVC, FEV_1_, and FEV_1_ to FVC ratio) were first adjusted for age and height (model 1), then additionally for weight, marital status, and education (model 2), and then also for the variables in model 2 and additionally for smoking and use of water pipe (model 3). The %-predicted values as outcomes were analyzed similarly, but the models were not adjusted for age and height, as these are already included in the predicted values.

## Results

The hairdressers had a longer average duration of work, measured in years, and longer weekly working hours, were more often married, had lower level of education, smoked more often and used water pipe more often, than the office workers as the reference group (Table 1).

**Table 1:** Characteristics of the study population

Variable	Hairdressers (Exposed group) (*n* = 140)	Office workers (unexposed reference group) (*n* = 140)	*p* value
Age (years), mean ± SD	34.40 ± 8.40	34.40 ± 5.70	0.95^a^
Height (cm), mean ± SD	160.20 ± 6.10	161.20 ± 5.90	0.18^a^
Weight (kg), mean ± SD	63.49 ± 8.97	64.03 ± 9.57	0.62^a^
BMI (kg/m^2^), mean ± SD	24.73 ± 3.33	24.67 ± 3.63	0.87^a^
Work duration (years), mean ± SD	11.12 ± 7.58	8.48 ± 5.66	0.001^a^
Work duration (years), median (Q1–Q3)	10 (6–15)	8 (5–11)	0.003^b^
Working hours per week, mean ± SD	45.72 ± 11.45	39.04 ± 9.25	< 0.0001^a^
Working hours per week, median (Q1–Q3)	48 (42–54)	40 (40–44)	< 0.0001^b^
Married, *n* (%)	95 (67.90)	57 (40.70)	< 0.0001^c^
Education, *n* (%)			< 0.0001^b^
Elementary	14 (10)	0 (0.0)	
High school	97 (69.30)	76 (54.30)	
Upper high school	29 (20.70)	64 (45.70)	
Smoking, *n* (%)	6 (4.30)	0 (0.0)	0.03^c^
Water pipe, *n* (%)	39 (27.90)	2 (1.40)	< 0.0001^d^

^a^Independent samples *t* test

^b^Mann–Whitney *U* test

^c^Fisher’s exact test

^d^Chi-square test

Regarding the use of precautionary safety measures in their work environment, we found that only 57% of hairdressers used a protective mask, but almost all of them (94%) used gloves. This difference could be attributed to the discomfort associated with wearing a facial mask for prolonged periods, especially in a warm climate (data not shown). Furthermore, 66% of the hairdressers worked in a space with air conditioning Table S1 shows the effect of the presence of air conditioning on the absolute and %-predicted values of lung function parameters. The presence of air conditioning was related to higher levels of VC, FEV1, and FVC, but a lower level of FEV1/FVC. There were no differences in the prevalence of respiratory symptoms.

In this study, 38% of hairdressers had completed some safety training. However, only 5% of them had regular health checkups arranged. The prevalence of any lung function deficits was 75% (*n* = 105) in the exposed and 21% (*n* = 30) in the reference group. All the lung function deficits were more common among the exposed compared to the unexposed group. Restriction (59%), followed by obstruction (11%), and obstruction with restriction (5%) are the majority of the impairments among the exposed group, respectively (Table 2). The exposed group had consistently a higher prevalence of all respiratory symptoms and these were statistically significantly increased (apart from phlegm production and productive cough) compared to the unexposed, even after adjusting for potential confounders (Table 3). In the fully adjusted models, the PR (95% CI) was 2.18 (1.26–3.77) for cough, 9.59 (1.004–91.73) for wheezing, 2.06 (1.25–3.39) for shortness of breath, and 3.31 (1.84–5.97) for tightness in the chest. A dose–response relation between the cumulative work duration and the risk of respiratory symptoms was also observed (Table 3). For all the symptoms, the risk was lowest among those with the shortest work duration (< 6 years), and then increased with an increasing work duration. However, for the very longest work duration (> 15 years), there was a plateau-effect in the risk of cough, phlegm production and productive cough, showing a slightly lower risk related to the longest work duration (> 15 years). In addition, more than half of the participating hairdressers (53%) reported having experienced skin problems (data not shown).

**Table 2:** Prevalence of lung function deficits

Type of deficit	Exposed group (*N* = 140)	Unexposed group (*N* = 140)*
Obstruction^a^	15 (10.71%)	9 (6.47%)
Restriction^b^	83 (59.29%)	18 (12.95%)
Obstruction with restriction^c^	7 (5.00%)	2 (1.44%)
Normal^d^	35 (25.00%)	110 (79.14%)

^a^FVC normal (≥ 80%), FEV_1_ reduced (< 80%), FEV_1_/FVC reduced (< 0.7)

^b^FVC reduced (< 80%), FEV_1_ normal or reduced (≥ 80% OR < 80%), FEV_1_/FVC normal or increased (≥ 0.7)

^c^FVC reduced (< 80%), FEV_1_ reduced (< 80%), FEV_1_/FVC reduced (< 0.7)

^d^FVC normal (≥ 80%), FEV_1_ normal (≥ 80%), FEV_1_/FVC normal (≥ 0.7)

^*^One value is missing for one participant in the reference group

**Table 3:** Effect of exposure on respiratory symptoms

Symptom	Exposure	*n* (%)	Unadjusted model PR (95% CI)	Adjusted model 1^a^ PR (95% CI)	Adjusted model 2^b^ PR (95% CI)
Cough	Office workers *N* = 140	24 (17.14)	1.00		
	Hairdressers *N* = 140	59 (42.14)	**2.46 (1.53–3.95)**	**2.44 (1.46–4.09)**	**2.18 (1.26–3.77)**
	Work duration ≤ 6 years, *N* = 43	14 (32.56)	1.90 (0.98–3.67)	1.77 (0.85–3.71)	1.64 (0.76–3.50)
	Work duration > 6 and ≤ 10 years, *N* = 35	16 (45.71)	**2.67 (1.42–5.02)**	**2.59 (1.33–5.05)**	**2.31 (1.16–4.61)**
	Work duration > 10 and ≤ 15 years, *N* = 35	19 (54.29)	**3.17 (1.73–5.78)**	**3.23 (1.69–6.18)**	**2.86 (1.46–5.63)**
	Work duration > 15 years, *N* = 27	10 (37.04)	**2.16 (1.03–4.52)**	2.29 (1.00–5.22)	1.98 (0.84–4.64)
Phlegm	Office workers *N* = 140	19 (13.57)	1.00		
	Hairdressers *N* = 140	30 (21.43)	1.58 (0.89–2.81)	1.84 (0.98–3.45)	1.77 (0.91–3.47)
	Work duration ≤ 6 years, *N* = 43	6 (13.95)	1.03 (0.41–2.57)	0.89 (0.32–2.45)	0.92 (0.32–2.60)
	Work duration > 6 and ≤ 10 years, *N* = 35	6 (17.14)	1.26 (0.50–3.16)	1.23 (0.47–3.21)	1.20 (0.45–3.21)
	Work duration > 10 and ≤ 15 years, *N* = 35	13 (37.14)	**2.74 (1.35–5.54)**	**3.89 (1.81–8.39)**	**3.76 (1.66–8.51)**
	Work duration > 15 years, *N* = 27	5 (18.51)	1.36 (0.51–3.65)	2.47 (0.83–7.30)	2.32 (0.76–7.07)
Productive cough	Office workers *N* = 140	17 (12.14)	1.00		
	Hairdressers *N* = 140	23 (16.43)	1.35 (0.72–2.53)	1.41 (0.71–2.81)	1.27 (0.60–2.68)
	Work duration ≤ 6 years, *N* = 43	4 (9.30)	0.77 (0.26–2.28)	0.58 (0.17–1.94)	0.56 (0.16–1.94)
	Work duration > 6 and ≤ 10 years, *N* = 35	7 (20.00)	1.65 (0.68–3.97)	1.51 (0.60–3.82)	1.37 (0.52–3.59)
	Work duration > 10 and ≤ 15 years, *N* = 35	8 (22.86)	1.88 (0.81–4.36)	2.24 (0.90–5.55)	2.03 (0.78–5.28)
	Work duration > 15 years, *N* = 27	4 (14.81)	1.22 (0.41–3.63)	1.84 (0.54–6.19)	1.55 (0.43–5.55)
Wheezing	Office workers *N* = 140	1 (0.71)	1.00		
	Hairdressers *N* = 140	9 (6.43)	**9 (1.14–71.04)**	**9.81 (1.09–88.36)**	**9.59 (1.004–91.73)**
	Work duration ≤ 6 years, *N* = 43	0 (0.00)	NA	NA	NA
	Work duration > 6 and ≤ 10 years, *N* = 35	1 (2.86)	4 (0.25–63.95)	3.65 (0.21–62.31)	4 (0.22–71.65)
	Work duration > 10 and ≤ 15 years, *N* = 35	3 (8.57)	**12 (1.25–115.36)**	**15.71 (1.45–169.80)**	**17.54 (1.56–197.26)**
	Work duration > 15 years, *N* = 27	5 (18.52)	**25.93 (3.03–221.91)**	**48.72 (4.46–532.40)**	**42 (3.46–510.26)**
Shortness of breath	Office workers *N* = 140	30 (21.43)	1.00		
	Hairdressers *N* = 140	66 (47.14)	**2.20 (1.43–3.39)**	**2.20 (1.37–3.52)**	**2.06 (1.25–3.39)**
	Work duration ≤ 6 years, *N* = 43	13 (30.23)	1.41 (0.74–2.70)	1.28 (0.63–2.63)	1.23 (0.59–2.57)
	Work duration > 6 and ≤ 10 years, *N* = 35	17 (48.57)	**2.27 (1.25–4.11)**	**2.12 (1.14–3.95)**	**1.98 (1.04–3.77)**
	Work duration > 10 and ≤ 15 years, *N* = 35	19 (54.29)	**2.53 (1.43–4.50)**	**2.63 (1.42–4.86)**	**2.47 (1.31–4.68)**
	Work duration > 15 years, *N* = 27	17 (62.96)	**2.94 (1.62–5.33)**	**3.40 (1.71–6.74)**	**3.12 (1.54–6.33)**
Chest tightness	Office workers *N* = 140	19 (13.57)	1.00		
	Hairdressers *N* = 140	58 (41.43)	**3.05 (1.82–5.12)**	**3.53 (2.01–6.19)**	**3.31 (1.84–5.97)**
	Work duration ≤ 6 years, *N* = 43	13 (30.23)	**2.23 (1.10–4.51)**	**2.40 (1.09–5.27)**	**2.40 (1.07–5.34)**
	Work duration > 6 and ≤ 10 years, *N* = 35	15 (42.86)	**3.16 (1.60–6.21)**	**3.45 (1.69–7.06)**	**3.23 (1.55–6.75)**
	Work duration > 10 and ≤ 15 years, *N* = 35	17 (48.57)	**3.58 (1.86–6.89)**	**4.32 (2.14–8.70)**	**4.05 (1.95–8.38)**
	Work duration > 15 years, *N* = 27	13 (48.15)	**3.55 (1.75–7.18)**	**4.39 (1.97–9.74)**	**3.93 (1.72–8.96)**

The statistically significant effects are shown bolded

^a^Adjusted for age, marital status, education, and BMI

^b^Adjusted for age, marital status, education, BMI, smoking, and use of water pipe

Table 4 shows the effect of the exposure on the absolute pulmonary function levels and Table 5 on the %-predicted measures. Both the absolute and the %-predicted VC, FVC, and FEV_1_ levels were reduced among the hairdressers compared to the office workers. In the fully adjusted model, the VC was on average 359 ml (95% CI 493 ml to 226 ml), the FVC 639 ml (95% CI 763 ml to 514 ml), and the FEV_1_ 536 ml (95% CI 653 ml to 419 ml) lower among the hairdressers compared to the reference group. There was no significant effect on FEV_1_/FVC. A dose–response effect related to cumulative work duration was also found on the lung function measures (Tables 4 and 5). There was a significant reduction of the absolute and the %-predicted VC, FVC, and FEV_1_ by each year of increasing work duration. In addition, the lung function measures decreased in relationship to work duration categorized into quartiles, from the shortest (< 6 years) up to those with reasonably long work duration (> 10 and ≤ 15 years), then having slightly smaller decrease in the very long work duration category (> 15 years).

**Table 4:** Effect of exposure on the absolute lung function parameters

Lung function parameter	Exposure	Unadjusted model *β* (95% CI)	Adjusted model 1^a^ *β* (95% CI)	Adjusted model 2^b^ *β* (95% CI)	Adjusted model 3^c^ *β* (95% CI)
VC, L	Office workers *N* = 140	Baseline level			
	Hairdressers *N* = 140	− **0.45 (− 0.57 to − 0.32)**	**− 0.42 (− 0.53 to − 0.31)**	**− 0.38 (− 0.51 to − 0.26)**	**− 0.36 (− 0.49 to − 0.22)**
	Work duration, per year	**− 0.03 (− 0.04 to − 0.02)**	**− 0.03 (− 0.03 to − 0.02)**	**− 0.02 (− 0.03 to − 0.01)**	**− 0.021 (− 0.03 to − 0.01)**
	Work duration ≤ 6 years, *N* = 43	**− 0.25 (− 0.43 to − 0.07)**	**− 0.26 (− 0.43 to − 0.088)**	**− 0.20 (− 0.39 to − 0.02)**	− 0.12 (− 0.38 to 0.003)
	Work duration > 6 and ≤ 10 years, *N* = 35	**− 0.43 (− 0.62 to − 0.23)**	**− 0.47 (− 0.65 to − 0.29)**	**− 0.44 (− 0.62 to − 0.26)**	**− 0.41 (− 0.60 to − 0.22)**
	Work duration > 10 and ≤ 15 years, *N* = 35	**− 0.62 (− 0.81 to − 0.43)**	**− 0.56 (− 0.74 to − 0.38)**	**− 0.52 (− 0.71 to − 0.34)**	**− 0.50 (− 0.70 to − 0.31)**
	Work duration > 15 years, *N* = 27	**− 0.58 (− 0.79 to − 0.36)**	**− 0.42 (− 0.64 to − 0.21)**	**− 0.38 (− 0.60 to − 0.16)**	**− 0.35 (− 0.58 to − 0.12)**
FEV_1_, L	Office workers *N* = 140	Baseline level			
	Hairdressers *N* = 140	**− 0.57 (− 0.69 to − 0.46)**	**− 0.55 (− 0.65 to − 0.45)**	**− 0.54 (− 0.65 to − 0.43)**	**− 0.53 (− 0.65 to − 0.42)**
	Work duration, per year	**− 0.03 (− 0.04 to − 0.03)**	**− 0.03 (− 0.04 to − 0.02)**	**− 0.03 (− 0.04 to − 0.023)**	**− 0.03 (− 0.04 to − 0.02)**
	Work duration ≤ 6 years, *N* = 43	**− 0.38 (− 0.54 to − 0.22)**	**− 0.43 (− 0.58 to − 0.27)**	**− 0.37 (− 0.53 to − 0.21)**	**− 0.37 (− 0.54 to − 0.21)**
	Work duration > 6 and ≤ 10 years, *N* = 35	**− 0.56 (− 0.73 to − 0.38)**	**− 0.61 (− 0.77 to − 0.45)**	**− 0.63 (− 0.78 to − 0.47)**	**− 0.62 (− 0.79 to − 0.46)**
	Work duration > 10 and ≤ 15 years, *N* = 35	**− 0.72 (− 0.90 to − 0.55)**	**− 0.66 (− 0.82 to − 0.50)**	**− 0.66 (− 0.82 to − 0.50)**	**− 0.66 (− 0.83 to − 0.49)**
	Work duration > 15 years, *N* = 27	**− 0.71 (− 0.90 to − 0.52)**	**− 0.52 (− 0.71 to − 0.33)**	**− 0.50 (− 0.02 to − 0.01)**	**− 0.50 (− 0.70 to − 0.30)**
FVC, L	Office workers *N* = 140	Baseline level			
	Hairdressers *N* = 140	**− 0.68 (− 0.80 to − 0.56)**	**− 0.65 (− 0.75 to − 0.545)**	**− 0.63 (− 0.75 to − 0.51)**	**− 0.64 (− 0.76 to − 0.51)**
	Work duration, per year	**− 0.04 (− 0.05 to − 0.03)**	**− 0.04 (− 0.04 to− 0.03)**	**− 0.03 (− 0.04 to − 0.02)**	**− 0.03 (− 0.04 to − 0.02)**
	Work duration ≤ 6 years, *N* = 43	**− 0.49 (− 0.66 to − 0.32)**	**− 0.51 (− 0.68 to − 0.35)**	**− 0.46 (− 0.63 to − 0.28)**	**− 0.47 (− 0.64 to − 0.29)**
	Work duration > 6 and ≤ 10 years, *N* = 35	**− 0.60 (− 0.79 to − 0.41)**	**− 0.65 (− 0.82 to − 0.49)**	**− 0.66 (− 0.83 to − 0.49)**	**− 0.67 (− 0.85 to − 0.50)**
	Work duration > 10 and ≤ 15 years, *N* = 35	**− 0.90 (− 1.09 to − 0.71)**	**− 0.83 (− 1 to − 0.67)**	**− 0.82 (− 1 to − 0.65)**	**− 0.84 (− 1.01 to − 0.66)**
	Work duration > 15 years, *N* = 27	**− 0.80 (− 1.01 to − 0.59)**	**− 0.61 (− 0.81 to − 0.41)**	**− 0.59 (− 0.79 to − 0.38)**	**− 0.60 (− 0.81 to − 0.38)**
FEV_1_: FVC, %	Office workers *N* = 140	Baseline level			
	Hairdressers *N* = 140	0.64 (− 1.79 to 3.06)	0.55 (− 1.88 to 2.98)	0.47 (− 2.23 to 3.17)	0.85 (− 2.02 to 3.73)
	Work duration, per year	− 0.07 (− 0.23 to 0.08)	− 0.04 (− 0.21 to 0.11)	− 0.07 (− 0.25 to 0.12)	− 0.05 (− 0.24 to 0.14)
	Work duration ≤ 6 years, *N* = 43	2.01 (− 1.53 to 5.54)	1.23 (− 2.54 to 5.01)	1.54 (− 2.51 to 5.58)	1.74 (− 2.41 to 5.90)
	Work duration > 6 and ≤ 10 years, *N* = 35	− 0.83 (− 4.67 to 3.01)	− 1.17 (− 5.06 to 2.72)	− 1.37 (− 5.37 to 2.64)	− 0.89 (− 5.02 to 3.23)
	Work duration > 10 and ≤ 15 years, *N* = 35	1.98 (− 1.85 to 5.82)	2.24 (− 1.64 to 6.12)	2.12 (− 1.96 to 6.21)	2.45 (− 1.77 to 6.68)
	Work duration > 15 years, *N* = 27	− 1.38 (− 5.64 to 2.88)	− 0.46 (− 5.13 to 4.21)	− 0.49 (− 5.34 to 4.362)	0.10 (− 4.87 to 5.07)

The statistically significant effects are shown bolded

^a^Adjusted for age and height

^b^Adjusted for age, height, weight, marital status, and education

^c^Adjusted for age, height, weight, marital status, education, smoking, and use of water pipe

**Table 5:** Effect of exposure on the percentage predicted lung function parameters

Lung function parameter	Exposure	Unadjusted model *β* (95% CI)	Adjusted model 1^a^ *β* (95% CI)	Adjusted model 2^b^ *β* (95% CI)
VC, %-predicted	Office workers *N* = 140	Baseline level		
	Hairdressers *N* = 140	− **11.22 (− 14.82 to − 7.62)**	**− 10.61 (− 14.55 to − 6.66)**	**− 9.94 (− 14.15 to − 5.72)**
	Work duration, per year	**− 0.52 (− 0.77 to − 0.28)**	**− 0.42 (− 0.68 to − 0.15)**	**− 0.36 (− 0.63 to − 0.10)**
	Work duration ≤ 6 years, *N* = 43	**− 7.96 (− 13.18 to − 2.74)**	**− 7.40 (− 12.87 to − 1.94)**	**− 6.97 (− 12.64 to − 1.31)**
	Work duration > 6 and ≤ 10 years, *N* = 35	**− 13.74 (− 19.40 to − 8.08)**	**− 13.52 (− 19.26 to − 7.78)**	**− 12.72 (− 18.67 to − 6.76)**
	Work duration > 10 and ≤ 15 years, *N* = 35	**− 14.97 (− 20.63 to − 9.32)**	**− 14.03 (− 20 to − 8.08)**	**− 13.57 (− 19.71 to − 7.42)**
	Work duration > 15 years, *N* = 27	**− 8.27 (− 14.56 to − 1.97)**	− 6.58 (− 13.28 to 0.11)	− 5.79 (− 12.60 to 1.03)
FEV_1_, %-predicted	Office workers *N* = 140^c^	Baseline level		
	Hairdressers *N* = 140	**− 15.78 (− 19.85 to − 11.71)**	**− 14.38 (− 18.85 to − 9.91)**	**− 14.28 (− 19.07 to − 9.50)**
	Work duration, per year	**− 0.76 (− 1.04 to − 0.49)**	**− 0.62 (− 0.92 to − 0.32)**	**− 0.58 (− 0.88 to − 0.27)**
	Work duration ≤ 6 years, *N* = 43	**− 12.13 (− 18.04 to − 6.22)**	**− 9.83 (− 16 to − 3.68)**	**− 9.91 (− 16.34 to − 3.49)**
	Work duration > 6 and ≤ 10 years, *N* = 35	**− 18.42 (− 24.82 to − 12.02)**	**− 18.33 (− 24.81 to − 11.85)**	**− 18.19 (− 24.94 to − 11.45)**
	Work duration > 10 and ≤ 15 years, *N* = 35	**− 19.76 (− 26.16 to − 13.36)**	**− 18.31 (− 25.03 to − 11.58)**	**− 18.37 (− 25.33 to − 11.41)**
	Work duration > 15 years, *N* = 27	**− 13 (− 20.12 to − 5.88)**	**− 10.41 (− 17.97 to − 2.85)**	**− 10.20 (− 17.92 to − 2.47)**
FVC, %-predicted	Office workers *N* = 140	Baseline level		
	Hairdressers *N* = 140	**− 16.73(− 20.49 to − 12.97)**	**− 15.39 (− 19.52 to − 11.27)**	**− 15.72 (− 20.15 to − 11.30)**
	Work duration, per year	**− 0.77 (− 1.02 to− 0.50)**	**− 0.61 (− 0.89 to − 0.32)**	**− 0.57 (− 0.86 to − 0.28)**
	Work duration ≤ 6 years, *N* = 43	**− 13.41 (− 18.85 to − 7.98)**	**− 11.55 (− 17.23 to − 5.86)**	**− 12.02 (− 17.93 to − 6.10)**
	Work duration > 6 and ≤ 10 years, *N* = 35	**− 18.03 (− 23.92 to − 12.14)**	**− 17.83 (− 23.80 to − 11.86)**	**− 18.20 (− 24.41 to − 11.98)**
	Work duration > 10 and ≤ 15 years, *N* = 35	**− 22.28 (− 28.17 to − 16.39)**	**− 20.82 (− 27.01 to − 14.62)**	**− 21.28 (− 27.69 to − 14.87)**
	Work duration > 15 years, *N* = 27	**− 13.11 (− 19.67 to − 6.56)**	**− 10.62 (− 17.58 to − 3.65)**	**− 10.85 (− 17.96 to − 3.73)**
FEV_1_: FVC, %-predicted	Office workers *N* = 140^d^	Baseline level		
	Hairdressers *N* = 140	0.25 (− 3.61 to 4.11)	0.58 (− 3.69 to 4.85)	0.84 (− 3.73 to 5.42)
	Work duration, per year	− 0.004 (− 0.25 to 0.24)	0.007 (− 0.27 to 0.28)	0.01 (− 0.26 to 0.30)
	Work duration ≤ 6 years, *N* = 43	0.20 (− 5.45 to 5.85)	0.99 (− 4.97 to 6.97)	1.15 (− 5.06 to 7.37)
	Work duration > 6 and ≤ 10 years, *N* = 35	− 1.64 (− 7.77 to 4.48)	− 1.68 (− 7.95 to 4.58)	− 1.32 (− 7.85 to 5.20)
	Work duration > 10 and ≤ 15 years, *N* = 35	2.41 (− 3.71 to 8.54)	2.76 (− 3.74 to 9.26)	2.94 (− 3.79 to 9.67)
	Work duration > 15 years, *N* = 27	− 0.04 (− 6.85 to 6.77)	0.72 (− 6.59 to 8.04)	1.09 (− 6.37 to 8.57)

The statistically significant effects are shown bolded

^a^Adjusted for weight, marital status, and education

^b^Adjusted for weight, marital status, education, smoking, and use of water pipe

^c^FEV_1_, %-predicted missing for one participant in the reference group

^d^FEV_1_: FVC, %-predicted missing for on participant in the reference group

## Discussion

In this cross-sectional study of hairdressers as the exposed group and office workers as the unexposed reference group from Shiraz, Iran, we showed that the hairdressers had significantly increased risks of respiratory symptoms, including cough, wheezing, shortness of breath and tightness in the chest, as well as significantly decreased VC, FVC, and FEV_1_ levels. Furthermore, increasing duration of work as a hairdresser was related to an increase in the risk of respiratory symptoms and a decrease in the lung function levels, which demonstrated a dose–response relationship. Interestingly, for cough, phlegm production, productive cough, and tightness in the chest, and for the lung function measures, we observed a plateau-effect among those with the longest duration of work as a hairdresser. A healthy worker effect is likely to contribute to this observation. This means that those hairdressers who stay for the longest in this profession may be selected to be somewhat more ‘resistant’ against the development of symptoms and/or lung function deficits as a response to the exposures, which would affect the values in the group with the longest exposure period. In contrast, the risk of wheezing and shortness of breath was the highest among those with the longest work duration.

### Synthesis with previous knowledge

Consistent findings of increased risk of respiratory symptoms in hairdressers have been reported from several high-income countries (Brisman et al. 2003; Hollund et al. 2001; Leino et al. 1997), from Turkey (Akpinar-Elci), but in only one previous study from Iran (Hashemi et al. 2010). Our findings extend the existing knowledge to a new geographic and cultural setting. Furthermore, the significant decrease of FVC level in hairdressers compared to the reference group (Table 3) with relatively well-preserved FEV_1_/FVC ratio, suggests the presence of an early restrictive lung function process. There is evidence that hairsprays and hair dyes increase the risk of idiopathic pulmonary fibrosis, which is characterized by restrictive lung function process (Awadalla et al. 2012; Nagata et al. 1997). A cross-sectional study from Finland reported a higher odds ratio for cough (1.4, 95% CI 1.1–1.9), dyspnea (1.5, 95% CI 1–2.2), and dyspnea with cough (1.6, 95% CI 1.0–2.7) in hairdressers compared to saleswomen (Leino et al. 1997). A cross-sectional study carried out in Larissa, central Greece (Skoufi et al. 2013), evaluated associations between occupational exposures and lung function levels as well as reported symptoms in a group of 94 hairdressers compared to a reference group of 39 office workers. Hairdressers reported more severe dyspnea (*p* = 0.03) and eye (*p* = 0.001) and throat (*p* = 0.007) irritation compared to the reference group. Lower FEV_1_/FVC (*p* < 0.001) was observed among hairdressers, suggesting obstructive lung function impairment. A larger working area and presence of window ventilation were associated with better lung function levels. In a population-based cross-sectional study of 184 hairdressers in Izmir, Turkey, potential risk factors of occupational asthma among hairdressers were evaluated (Akpinar-Elci et al. 2002). Dry cough was observed with a prevalence of 8.7% (*n* = 16), dyspnea with a prevalence of 3.8% (*n* = 7), chest tightness with a prevalence of 3.3% (*n* = 6), wheezing with a prevalence of 3.3% (*n* = 6) and occupational asthma with a prevalence of 14.6% (*n* = 25) among hairdressers. The authors reported a significant relation between work intensity in hairdressing and risk of asthma (Akpinar-Elci et al. 2002). All the spirometry tests, including FEV_1_ and FEV_1_/FVC, were above 80% of predicted values (Akpinar-Elci et al. 2002). In a cohort study of 161 female hairdressers in Hebron city, Palestine, changes in self-reported respiratory symptoms over 5 years, as well as to the lung function decline were assessed (Nemer et al. 2015). Prevalence difference of chest tightness (PD = 0.037, 95% CI 0.005–0.069), shortness of breath (PD = 0.038, 95% CI 0.001–0.076), and morning phlegm (PD = 0.068, 95% CI 0.020–0.115) were significantly higher among the exposed group when compared to their baseline situation. Those hairdressers who quit reported a non-significant decrease in symptom occurrence. Statistically significant decreases in FVC of 35 ml/year (95% CI 26–44 mL/year) during the follow-up and in FEV_1_ of 31 ml/year (95% CI 25–36 ml/year) were found in hairdressers remaining active. In another cross-sectional study from Iran, the occurrence of all respiratory symptoms, including cough, breathless, wheezing, and phlegm production were significantly higher in the 50 hairdressers compared to the reference group of 50 workers selected randomly from the general population (Hashemi et al 2010). FVC and FEV_1_ were significantly lower in the hairdressers than in the reference group. Almost 30% of the hairdressers had an FEV_1_ level < 80% of predicted. The bleaching powder and hair sprays were found to be the most irritant chemicals that were suggested to provoke the respiratory symptoms.

Thus, our findings of increased respiratory symptoms in combination with restrictive lung function deficit among workers in hairdressing salons provide evidence of hazardous working conditions in such salons in Iran. These findings are consistent with previous international studies from other parts of the world, apart that we found restrictive rather than obstructive pattern of lung function deficits. Our cross-sectional findings raise the important study question that hairdressing exposures may contribute to a restrictive process in lung function, and that lack of air conditioning seems to enhance such adverse effects. These findings may be generalizable to other parts of the world with warm climate conditions. Hairdressers are exposed to numerous reactive agents with potential irritant, inflammatory, and sensitizing effects. Ammonium compounds, and potassium and sodium persulfates as oxidizing agents have been previously reported to be specific agents of concern for hairdressers’ health. Especially, persulfate has been previously observed to be a rather common cause of occupational asthma and hypersensitivity-related respiratory symptoms in hairdressers (Moscato et al. 2005), while restriction detected in our study is likely to be related to a fibrotic process, possibly related to irritant mechanisms. Heat exposure has been suggested to augment the risks related to other indoor pollutants (McCormack et al. 2016; Rice et al. 2019), so it could explain why so strong health effects were observed in the warm climate in Iran and why lack of indoor ventilation was related to even larger adverse health effects. Iranian summers can be very hot, and for example, the average maximum temperature in July is 37 °C. The enhancement of the adverse effects in the absence of air conditioning may be an important finding that may be of importance also for other countries in the developing world.

The occupation-related adverse respiratory effects among hairdressers are likely to be mostly preventable, and thus, we recommend that primary preventive actions should be carried out in hairdressing saloons all over the world. As we found in this study, many basic precautionary measures, including training of hazards and recognizing the need for respiratory protection, are still not taken extensively. If facial masks are used because of working with hazardous products in the absence of specific ventilation, the type of protective equipment to be used is an important question. N95 respirators recommended by the US National Occupational Safety and Health Administration (OSHA) would provide protection against many particulates but provide little protection for vapors and gasses (OSHA 2012). Implementation of adequate ventilation systems in the hairdressing saloons could significantly decrease the exposure levels of airborne chemicals. Our data, which were based on a modified ATS respiratory questionnaire, did not address the adequacy of ventilation systems. However, we provide some evidence that only a small proportion of hairdressers had access to heat tempered air (i.e., there was no air conditioning). That is a concern that may be common in other low- and middle-income countries and present also in high-income countries.

A common approach to work-induced illness is relocation of the worker to a healthier work environment. However, it is unlikely that the hairdressing industry is able to empower workers with meaningful alternative placements in all areas of either high- or low-income countries. Efforts to replace products containing multiple hazardous substances with less harmful products should have priority (Lysdal et al. 2014), and a next logical step in preventing adverse health effects would be to develop safer products and to ensure their availability in Iran and other parts of the world.

Limitations of our study include unavailability of air measurements of workplace indoor air as well as sampling of products that were used. In addition, we were not able to use biomarkers to investigate potential presence of systemic inflammation in the airways or potential existence of chemical compounds in the body. Despite these limitations, we think these data are adequate to indicate that hairdressing exposures are hazardous in Iran and other similar areas of the world. Our results provide new evidence that absence of temperature conditioned air in hairdressing saloons increases the hazardous effects in such warm climates.

## Conclusions

Results of the current study provide additional evidence of significant relations between exposure to occupational chemical pollutants in hairdressing salons and increased prevalence of respiratory symptoms and decreased lung function levels. The pattern of lung function deficits suggests mainly restrictive lung process. This evidence indicates that insufficient attention has been paid to the occupational respiratory risks encountered in hairdressing work in the developing world. In Iran and many other countries in the area, only annual skin examinations have been organized. Among effective measures for mitigating these occupational risks among hairdressers are use of less harmful substances as well as emphasizing use of proper personal protective equipment and controlled engineering techniques to improve local air conditioning.

## Supplementary Information

Below is the link to the electronic supplementary material.

Supplementary file1 (DOCX 15 KB)

## Data Availability

All relevant data and materials are presented in the paper.
