# Supplementary material for: Occupational exposures and respiratory symptoms and lung function among hairdressers in Iran: a cross-sectional study
Source: Int Arch Occup Environ Health. 2021 Jan 18;94(5):877–87. doi: 10.1007/s00420-020-01645-z (PMC8238693; doi:10.1007/s00420-020-01645-z)
Supplement: Supplementary file 1 — Supplementary file1 (DOCX 15 KB) [file 420_2020_1645_MOESM1_ESM.docx]

**Table S1.** The association between the presence of air conditioning (yes = 1, no = 0) and the levels of lung function parameters among 140 hairdressers. The regression coefficients (β) represent differences of lung function parameters among the hairdressers with air conditioning (N=92) and without air conditioning (N=48). Adjustments made by linear regression analysis.

| Parameter | Unadjusted model  β (95% CI) | Adjusted model 1 ^a^  β (95% CI) | Adjusted model 2 ^b^  β (95% CI) | Adjusted model 3 ^c^  β (95% CI) | Adjusted model 4 ^d^  β (95% CI) |
| --- | --- | --- | --- | --- | --- |
| VC, L | **0.15 (0.007, 0.29)** | **0.159 (0.03, 0.29)** | **0.151 (0.02, 0.28)** | **0.15 (0.01, 0.28)** | **0.16 (0.02, 0.29)** |
| FEV_1_, L | 0.11 (-0.05, 0.27) | 0.113 (-0.04, 0.27) | 0.078 (-0.08, 0.24) | 0.08 (-0.08, 0.24) | 0.10 (-0.06, 0.26) |
| FVC, L | **0.23 (0.06, 0.39)** | **0.239 (0.08, 0.39)** | **0.2095 (0.05, 0.37)** | **0.22 (0.06, 0.38)** | **0.23 (0.07, 0.39)** |
| FEV_1_:FVC, % | -2.22 **(**-6.93**,** 2.48**)** | -2.36 **(**-7.11**,** 2.39**)** | -2.77 **(**-7.65**,** 2.09**)** | -2.93 **(**-7.86**,** 2.0**)** | -2.45 **(**-7.41**,** 2.52**)** |
| VC, %-predicted | 4.15 **(**-0.65**,** 8.96**)** | NA | 3.959 **(**-0.96**,** 8.88**)** | 3.76 **(**-1.18**,** 8.71**)** | 3.75 **(**-1.27**,** 8.76**)** |
| FEV_1_, %-predicted | 5.13 (-1.28, 11.55) | NA | 3.98 (-2.51, 10.47) | 4.11 (-2.48, 10.70) | 4.27 (-2.40, 10.94) |
| FVC, %-predicted | **7.17 (1.88, 12.47)** | NA | **6.282 (0.90, 11.66)** | **6.61 (1.14, 12.07)** | **6.58 (1.05, 12.12)** |
| FEV_1_:FVC, %-predicted | **-6.21 (-12.39, -0.04)** | NA | **-6.83 (-13.12, -0.55)** | **-7.06 (-13.45, -0.67)** | **-6.97 (-13.43, -0.52)** |

^a^ Adjusted for age (actual values) and height (actual values).

^b^ Adjusted for age (actual values), height (actual values), weight, marital status and education.

^c^ Adjusted for age (actual values), height (actual values), weight, marital status, education, smoking, and waterpipe.

^d^ Adjusted for age (actual values), height (actual values), weight, marital status, education, smoking, waterpipe, use of mask and work duration.
